# Association between changes in coronary artery circulation and cardiac venous retention: a lesson from cardiac computed tomography

**DOI:** 10.1007/s10554-012-0139-9

**Published:** 2012-10-18

**Authors:** Rafal Mlynarski, Agnieszka Mlynarska, Maciej Sosnowski

**Affiliations:** 1 Department of Electrocardiology, Upper-Silesian Medical Centre, ul Ziolowa 45/47, 40-635 Katowice, Poland; 2 Unit of Noninvasive Cardiovascular Diagnostics, Upper-Silesian Medical Centre, Katowice, Poland; 3 3rd Division of Cardiology, Medical University of Silesia, Katowice, Poland

**Keywords:** CABG, Coronary veins, Computed tomography, Coronary circulation

## Abstract

To use computed tomography (CT) image data to measure a potential association between the implantation of coronary artery bypass grafts (CABG) and changes in the coronary venous system has not yet been examined. In 112 (aged 59.4 ± 9.0; 45F) patients (pts.), a 64-slice CT angiography was performed. Patients were divided into 2 groups: CABG (56 pts.) and control (56 pts.)—without changes in coronaries. In each case, ten multi-planar reconstructions (MPR) and 3D volume rendering reconstructions using a 2 mm layer with ECG-gating, helical pitch: 12.8; rotation time: 0.4 s and average tube voltage: 135 kV at 380 mA. The visualization of the coronary veins was independently graded by 2 experts trained in CT. In the CABG group, the average number of visible coronary veins was 5.3 ± 1.3, while in the control group it was 3.1 ± 1.1 (*p* < 0.001). Statistical differences were also observed for the following coronary veins: posterolateral (control 2.1 ± 1.9 vs. CABG 2.9 ± 1.9; *p* < 0.05), lateral (control 2.2 ± 1.7 vs. CABG 3.1 ± 1.3; *p* < 0.01) and anterolateral (control 0.5 ± 0.9 vs. CABG 1.3 ± 1.0; *p* < 0.001). Implantation of CABG influences the coronary venous system. In patients after CABG, the number of identifiable coronary veins is significantly higher as compared to that in subjects without changes in coronaries. This might suggest an association between changes in coronary artery circulation and cardiac venous retention.

## Introduction

Knowledge of the importance of the coronary venous system has increased during recent decades mainly due to advances in electrophysiological procedures such as ablation or cardiac resynchronization [1–3]. In the past there was also an attempt to use the coronary venous system for the treatment of coronary artery disease (CAD); however, this method was not clinically appreciated [4].

It was shown that in some diseases like heart failure, CAD or myocardial infarction, the anatomy of the coronary veins was affected—this can be caused by changes in the flow. Information about changes in the venous anatomy is very important especially from an electrophysiologist’s point of view because many patients who qualify for resynchronization suffer from the diseases mentioned above.

We hypothesized that advanced CAD, in which the implantation of coronary bypass grafts (CABG) is necessary, can influence the coronary venous circulation and influence the venous anatomy. New imaging methods can be useful in finding an explanation for this phenomenon [5]. There have been several studies which have confirmed the usefulness of MSCT for the visualization of the coronary venous system, but none of them included patients with a previous bypass grafts implantation [6, 7].

The purpose of this study was to evaluate the cardiac venous anatomy in patients after coronary artery bypass graft surgery.

## Methods

112 patients (aged 59.4 ± 9.0; 45 females) in 2 equal groups were included into the trial:

- 56 patients with previously implanted bypass grafts (CABG group) in which MSCT was performed to check the patency of the grafts.
- 56 patients included due to a suspicion of CAD without changes within the coronaries (controls).

Patients were excluded if stenosis in the coronaries was present (controls), if they had atrial fibrillation, frequent premature heartbeats, renal insufficiency (serum creatinine > 1.2 mg/dl), hyperthyreosis, any known allergy to nonionic contrast agents or a previously implanted pacemaker with unipolar leads [9].

A 64-detector row cardiac computed tomography (CT) was performed on each patient. In all of the patients, the coronary venous system was analyzed including the number of visible coronary veins and the type of veins visualized (coronary sinus, anterior, antero-lateral, lateral, postero-lateral, posterior veins and middle cardiac veins). The nomenclature of those vessels was based on earlier published methodology [8].

Computed tomography was performed using an Aquilion 64 scanner (Toshiba Medical Systems, Japan). Scanning with retrospective ECG-gating was performed during a breath-hold using 64 slices with a collimated slice thickness of 0.5 mm. A breath-hold examination was performed in order to adjust the scanner settings.

The helical pitch was 12.8 (best mode) and the rotation time was 0.4 s. The tube voltage was strictly dependent on the patient’s body mass index (BMI): for BMI < 23.9, it was 120 kV at 330 mA, for BMI = 24.0–29.9, it was 135 kV at 380 mA and for BMI > 30.0, it was 135 kV at 430 mA. We used a pre-selected region of interest (ROI) in the descending aorta. Triggering started at 180 Hounsfield units. On average, 100 ml of non-ionic contrast agent (Ioperamid, Ultravist 370, Schering, Germany) was administered to each patient during the examination at an average rate of 4.5 ml/s in the control group and 130 ml at an average rate 4.5–05 ml/s in the CABG group in three phases.

The cut-off for heart rate (HR) was set at 65 bpm. If the HR was higher, metoprolol succinate (Betaloc, Astra Zeneca, Sweden) at a dosage of 5–10 mg was administered intravenously, unless contraindicated. If the expected HR slowing did not occur, the patient was excluded from the study. Sublingual nitroglycerin was not administered before image acquisition.

Reconstructions of data were performed on Vitrea 2 workstations (Vital Images, USA; software version 3.9.0.0 and 5.1). In each case, ten (10) axial image series and 3D volume rendering reconstructions were created from 0 to 90 % R–R intervals (step 10 %) using a 2.0 mm slice thickness to reduce the large amount of data.

Optimal phases were chosen based on the quality of the visualization using a scale that was developed and published by our team earlier (Table 1) [10].

**Table 1:** The scale used for the visualization of arteries and veins [10]

Score	Description
0	No vessel(s) present^a^
1^b^	Vessel, length less than 5 mm, weakly contrasted and/or with a number of artifacts
2	Between score 1 and score 3
3	Vessel more than 1 cm long, better contrasted. Sometimes areas not visible or artifacts occurred.
4	Between score 3 and score 5
5	Vessel well contrasted, clearly visible on the entire length of the vessel

^a^When a vein is not visualized in any of the phases, it has a grade of 0. However, this does not necessarily mean that the vein is absent—it could be e.g. very small and therefore lower than the resolution of the CT scanner

^b^Grade 1 means that a vessel is present but very poorly visualized

Vessels were graded by 2 experts trained in MSCT and experienced in the visualization of the coronary venous system (more than 250 venograms evaluated in MSCT). Reproducibility of the phase determination was evaluated using the Bland–Altman method and by the calculation of the inter-rater agreement coefficient kappa. Correlations between data were calculated by means of the Spearman rank coefficient (MedCalc Soft, Belgium).

The local Ethics Committee approved the study protocol. The study protocol complied with the version of the Helsinki Convention that was current at the time the study was designed.

## Results

The characteristics of the patients included are presented in Table 2.

**Table 2:** Characteristics of the patients included

	CABG	Control	*p*
Hemodynamic parameters^a^			
Ejection fraction (%)	58.1 ± 13.6	63.9 ± 7.9	0.0182
EDV (ml)	156.4 ± 41.6	135.5 ± 42.8	0.0375
ESV (ml)	71.7 ± 43.1	49.5 ± 21.9	0.003
Stroke volume (ml)	87.2 ± 20.3	83.2 ± 19.1	NS
Cardiac output (l/min)	5.4 ± 1.4	5.3 ± 1.9	NS
Myocardial mass (g)	150.6 ± 29.5	125.8 ± 41.4	0.0029
Myocardial volume (ml)	143.1 ± 28.3	113.4 ± 36.1	0.0003
Risk factors			
Heart rate (bpm)	62.2 ± 8.6	62.3 ± 15.5	NS
Hypercholesterolemia (%)	100	26.8	0.0001
Hypertension (%)	64.3	14.3	0.0003
Diabetes (%)	50	8.9	0.0004
Smoking (%)	35.7	21.4	NS

^a^Values by cardiac computer tomography

The number of visible cardiac veins was highly reproducible (inter-rater agreement kappa: 1.000; standard error: 0.000), while intra-observer agreement was excellent (mean difference 0, inter-rater agreement kappa 1.0).

Similarly, there was a very good agreement between observers in the evaluation of the quality of the reconstructions (95 % CI −0.945 to 1.000, inter-rater agreement kappa 0.974). There were similar results in the repeated evaluation of the score by the same observer (95 % CI 0.959–1.0000 and kappa 0.983).

A good quality visualization was obtained for both groups; however, the average quality of the visualization was statistically better in the CABG group (4.3 ± 0.8 points) as compared to the control group (3.9 ± 1.1, *p* < 0.01).

**Fig. 1:**
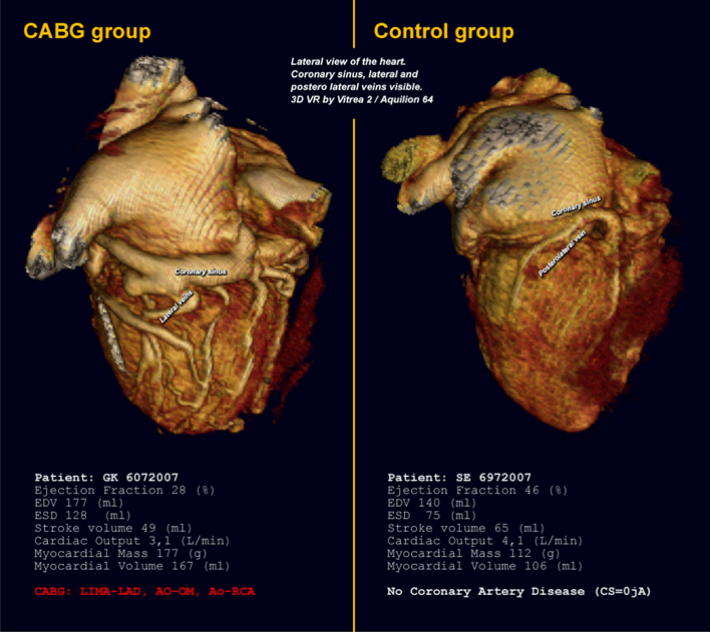
Example of the 3D anatomy of the heart in the *CABG* and *control* groups. Lateral view of the heart, 3D volume rendering reconstruction

**Fig. 2:**
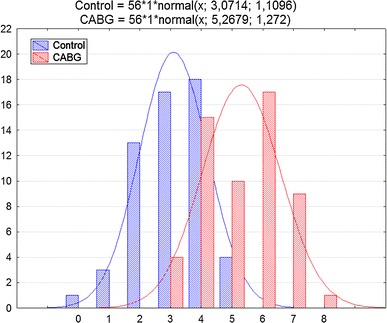
Dispositions of the occurrences of veins analyzed in the groups studied

The average number of coronary veins was 5.3 ± 1.3 in the CABG group, while in the control group, it reached 3.1 ± 1.1 (*p* < 0.001). Statistical differences were also observed for the following coronary veins: posterolateral, lateral and anterolateral. No statistical differences were observed for the radical veins: posterior vein and anterior vein. Relations in the number of coronary veins analyzed are presented in Table 3. An example of the anatomy from both groups is presented in Fig. 1. Additionally, the distribution of the occurrence of veins analyzed is presented in Fig. 2.

**Table 3:** Average number of coronary veins visualized in both groups

	Average ± SD		*p*
	CABG	Control	
Posterior vein	1.59 ± 1.86	1.30 ± 1.79	NS
Posterolateral vein	2.89 ± 1.86	2.05 ± 1.91	0.022
Lateral vein	3.14 ± 1.28	2.17 ± 1.65	0.019
Anterolateral vein	1.32 ± 0.97	0.51 ± 0.93	0.000
Anterior vein	2.28 ± 1.37	1.93 ± 1.47	NS

**Fig. 3:**
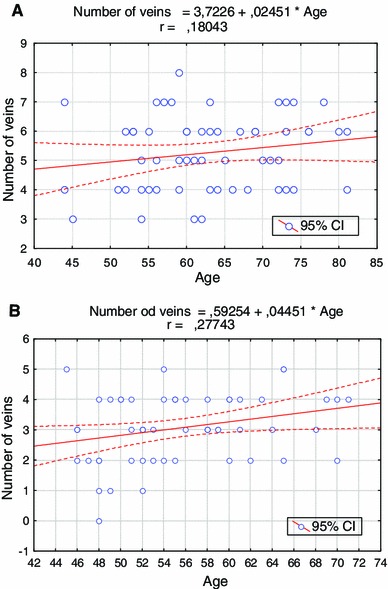
Correlations between age and the number of visible veins. **a** CABG group; **b** control group

We also observed that in older subjects the number of veins increased in both groups. A statistically significant correlation (r = 0.245; *p* < 0.05) was found only for control group. Such a correlation was not observed in the CABG group. Both correlations are presented graphically in Fig. 3.

## Discussion

To the best of our knowledge, there has been no research to date that shows the influence of cardiovascular pathologies on the anatomy of coronary veins. Because the role of the coronary venous system is very important as a “natural safety valve” in many diseases, the influence of any modification of coronary circulation into the coronary venous system has been the subject of some research and scientific hypotheses. More than 100 years ago, Pratt et al. presented the hypothesis of the perfusion of isolated feline hearts by the coronary venous system [4, 11]. Since that time, this hypothesis has been explored by Beck—anastomosis to arterial vessels and Lillehei et al. [12, 13]—using retroperfusion during cardiac surgery. This concept has not been fully utilized clinically because arterial surgical revascularization methods have been shown to be both safe and efficacious. On the other hand, many patients suffer from additional diseases such as heart failure or heart rhythm disorders where cardiac resynchronization (CRT) implantation or ablation is indicated. In such cases, cannulation of the coronary sinus or (in CRT) access to the coronary vein has to be performed. Our earlier studies regarding the visualization of the coronary venous system in MSCT documented the anatomy of coronary veins in subjects without changes in coronary arteries [8, 14]. Similar research has also been performed by other researchers [6, 15, 16]. During our clinical practice, we observed that in most patients qualified for CT coronary angiography for the evaluation of the patency of bypass grafts, the coronary venous system is more developed as compared to subjects without CAD. This observation contradicted information from electrophysiologists who found that patients have many problems after CABG when a left ventricle lead is implanted. Our results are in full agreement with the research of Hajaghaei et al. [17]. The authors showed a significant increase in the diameter of the coronary sinus in patients after CABG. They also confirmed that the coronary flow reserve (CFR) was significantly higher after cardiac surgery.

The results of this study might be explained by the fact that the implantation of CABG changes the distribution of pressures within the arterial and venous vessels. Higher pressure in the coronary venous system can cause the expansion of veins as a compensation mechanism. Also, one might hypothesize that increased coronary venous pressure can support the ischemic myocardium in the presence of advanced atherosclerotic lesions. Changes in contrast enhancement, as first proposed by Rybicki et al. [18], and then expanded upon by Steigner et al. [19] followed by Chow et al. [20] have been shown to contain additional diagnostic information. Our manuscript provides additional data to support the concept that coronary enhancement is related to flow, and more specifically that alterations in coronary blood flow patterns can be measured, at least in part, by CT. However, anatomo-pathology studies are necessary to confirm these explanations.

## Limitations of the study

Presented study is observational study only—we did not perform two examinations on each patient (before and after cardio surgery) due to the large dose of radiation. We are aware that the main aim of an MSCT examination in the included patients was the evaluation of the coronary arteries. Thus, as no special scan and contrast protocol for venous enhancement was used, in certain cases the existing veins could not be visualized. However, as the same protocol (arterial phase) was used in both groups, the observed differences in the venous anatomy are reliable. Our results do not explain why difficulties during implantation of CRT in patients after CABG occurred; we only documented that for some reason the number of coronary veins increases after bypasses. There is no other research describing the venous anatomy in patients after CABG; therefore we do not have the possibility to discuss these results with other authors.

## Conclusions

Advanced CAD where implantation of CABG is needed has a significant influence on the coronary venous system. The number of identifiable coronary veins is significantly higher in patients after CABG as compared to subjects without changes in coronaries. This might suggest an association between changes in coronary artery circulation and cardiac venous retention. The clinical significance and pathophysiological meaning of this finding requires further studies.
